# Mosquitoes in urban green spaces: using an island biogeographic approach to identify drivers of species richness and composition

**DOI:** 10.1038/s41598-017-18208-x

**Published:** 2017-12-19

**Authors:** Antônio Ralph Medeiros-Sousa, Aristides Fernandes, Walter Ceretti-Junior, André Barreto Bruno Wilke, Mauro Toledo Marrelli

**Affiliations:** Department of Epidemiology, School of Public Health, São Paulo University, Avenida Doutor Arnaldo 715, CEP 01246–904 São Paulo, Brazil

## Abstract

Mosquitoes are well known for their epidemiological importance as vectors of a wide range of human pathogens. Despite the many studies on medically important species, little is known about the diversity patterns of these insects in urban green spaces, which serve as shelter and refuge for many native and invasive species. Here, we investigate drivers of mosquito richness and composition in nine urban parks in the city of São Paulo, Brazil. Using the equilibrium theory of island biogeography, we tested predictive models for species richness and composition and performed nestedness analysis. We also investigated whether species loss tends to benefit vector mosquitoes. In the period 2011 to 2013, a total of 37,972 mosquitoes belonging to 73 species and 14 genera were collected. Our results suggest there is a species-area relationship, an increase in species similarity as richness is lost and a nested species composition pattern. Seven of the eight most commonly found species are considered vectors of human pathogens, suggesting a possible link between species loss and increased risk of pathogen transmission. Our data highlight the need for studies that seek to understand how species loss may affect the risk of infectious diseases in urban areas.

## Introduction

Mosquitoes (Diptera: Culicidae) are the most important disease-vector insects and are indirectly responsible for high morbidity and mortality in humans^1^. Mosquito-borne diseases, such as malaria, dengue fever, lymphatic filariasis, yellow fever and West Nile fever, are important causes of morbidity and mortality worldwide, especially in tropical and subtropical countries^2–6^.

While most mosquito species show a preference for specific types of larval habitats and are very sensitive to environmental changes, some tend to thrive in human-impacted environments, such as urban areas^7,8^. These highly impacted new ecosystems favor certain mosquito species that can achieve high abundances by breeding in artificial sites resulting from human activities. Examples of such mosquitoes are *Aedes aegypti* and *Culex quinquefasciatus*, which are responsible for transmitting pathogens that cause high morbidity to human populations in major cities around the world^3,4^.

It has been estimated that 54% of the human population lives in urban areas, a figure that is likely to increase in the coming decades. Currently, there are 28 “megacities” (cities with over 10 million inhabitants) in the world^9^. In major cities, vegetation tends to be heterogeneously distributed as fragmented “green islands” within the urban landscape^10^. Most of these green areas are urban parks designed to provide the population with a place where they can spend their leisure time, practice physical activities and have contact with nature. The creation and maintenance of such parks is one of the strategies adopted by the authorities in large cities to preserve natural habitats and biodiversity, as they serve as a shelter and refuge for many populations of native, migratory or introduced species^11,12^. Although several studies have sought to understand the responses of invertebrate groups to habitat fragmentation and isolation in urban green spaces^13,14^, little is known about mosquito diversity patterns in these spaces. Studies on mosquito diversity in urban green spaces can be useful in two ways: firstly, by elucidating the processes that lead to diversity patterns in urban ecosystems and, secondly, by allowing the role of biodiversity in reducing or increasing the risk of pathogen transmission to be investigated^15,16^.

Among the theories used to explain the regional processes that promote urban biodiversity, the equilibrium theory of island biogeography (ETIB), developed by MacArthur and Wilson^17^, has received strong support in studies of insect communities and other arthropods living in urban areas^14,18^. According to this theory, species richness on islands represents a dynamic equilibrium between immigration and extinction rates, which are affected by the size of the island and the distance to the source of colonization. As urban green fragments are different sizes, have different degrees of isolation and are separated by an environment that is inhospitable or less suitable for most species, the ETIB can be used as a conceptual framework to explain the diversity patterns in these fragments by treating them as island-like habitats^18,19^. One of the most important predictions of the ETIB concerns the relationship between patch size and species richness (the species-area relationship)^20,21^, as larger areas have higher habitat diversity, are larger targets for colonizers and support larger populations, making species less vulnerable to extinction. According to the ETIB, patch isolation may also influence species richness, since colonization decreases and extinction rates increase in more isolated fragments. Furthermore, the proximity between natural fragments can increase the chances of sustaining a meta-population, thereby reducing the risk of species extinction^14,18^.

The insular layout of urban green areas and variations in patch size and isolation can also be drivers for nested patterns, where species composition of small assemblages is a subset of species composition found in large assemblages^22^. Larger habitat patches support species with small and large minimum area requirements, while smaller patches only support those with small requirements^23,24^. Likewise, habitat isolation may limit colonization by species with low dispersal ability and favor colonization by those with greater dispersal ability^24,25^.

If mosquito assemblages found in urban green spaces follow patterns predicted by the ETIB, larger, less isolated fragments can be expected to have higher species richness than smaller, more isolated ones. In addition, the latter can be expected to have a subset of the species found in the species-richer fragments (i.e., a nested pattern), composed largely of a few ‘urban exploiters’ (invasive or native domiciled species). This would increase the vector-borne disease risk if there were a tendency for the most vector-competent species to be selectively favored and persist at the expense of biodiversity^26^.

**Figure 1:**
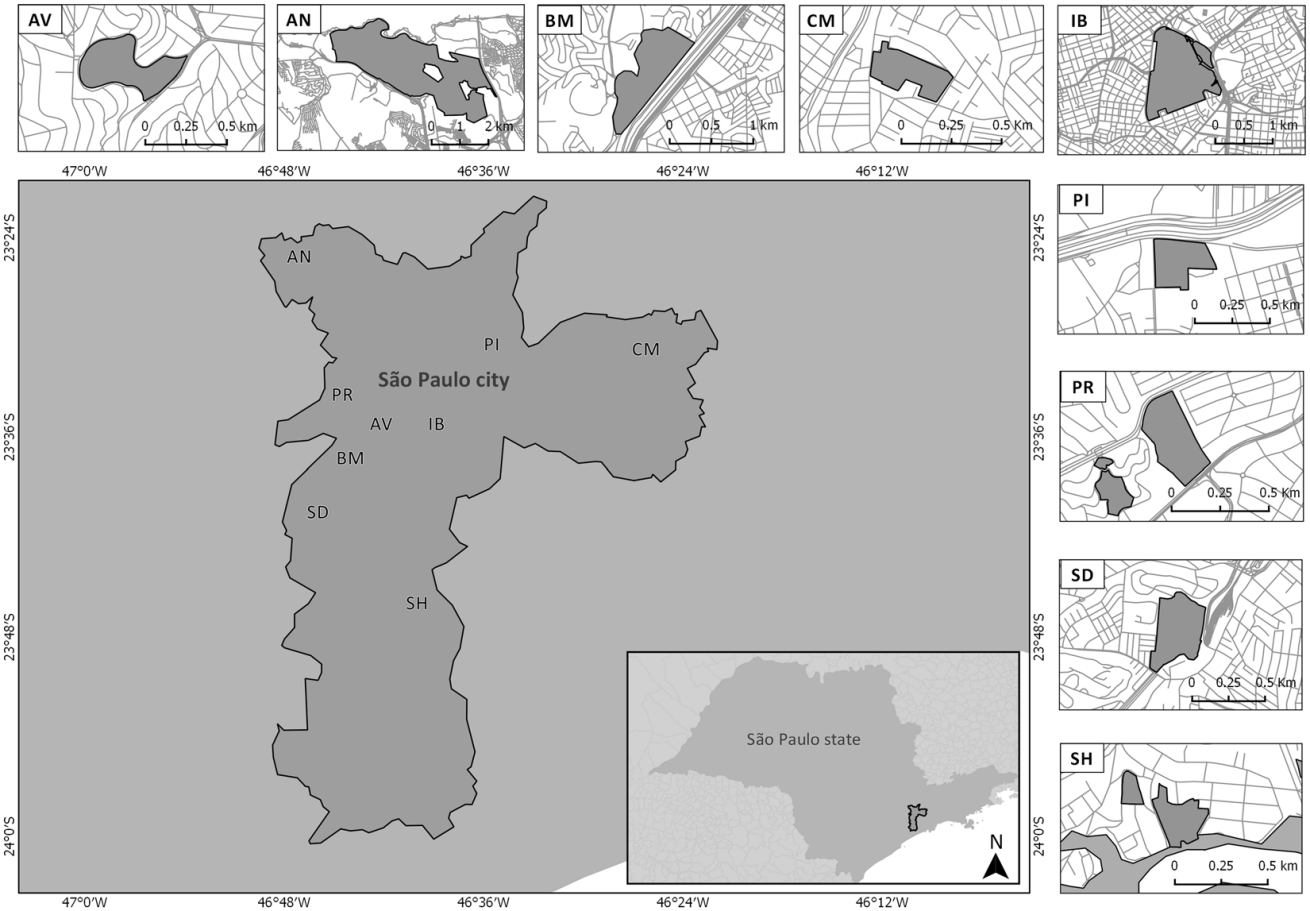
Map of the city of São Paulo showing the urban parks where the survey was conducted. AV - Alfredo Volpi, AN – Anhanguera, BM - Burle Marx, CM – Chico Mendes, IB – Ibirapuera, PI – Piqueri, PR – Previdência, SD – Santo Dias, SH – Shangrilá. This map was created using QGIS v2.18.9 (http://www.qgis.org).

Here, we used data on field collections and landscape analysis to investigate whether mosquito richness and composition in urban green areas may be predicted by the ETIB. We tested the hypotheses of a species-area-isolation relationship, increased species similarity between species-poor sites and a nested subset pattern. We also investigated whether species loss tends to benefit vector mosquitoes. The study was carried out from 2011 to 2013 in nine urban parks in the megacity of São Paulo (Fig. 1). The parks were selected to guarantee different fragment sizes and degrees of isolation (see Supplementary Table S1). Endemic dengue and emergent mosquito-borne diseases such as chikungunya and Zika fevers are serious public health issues in the city of São Paulo^27^, which is situated in the Brazilian Atlantic Forest biome, a biodiversity hotspot^28^ where hypoendemic malaria is present and several arboviruses transmitted by mosquitoes circulate enzootically in the wild, occasionally causing encephalitis and hemorrhagic fevers in humans^29,30^.

## Results

### Mosquito richness and composition

In all, 37,972 mosquitoes were collected, distributed in 73 species/taxa and 14 genera. Approximately two-thirds were 25,259 adult individuals, of which 21,075 were females. A total of 61 species were collected in the adult form and 41 in the immature form. Thirty-two species were found only in the adult form and 13 only in the immature form. The largest number of adult specimens was collected with Shannon traps (9,907) and the largest number of immatures with suction samples (9,474 mosquitoes), while the largest number of species was collected with the battery-powered aspirator (51 species) (see Supplementary Table S6 for further information). The five most abundant species, *Culex nigripalpus*, *Aedes albopictus*, *Cx*. *quinquefasciatus*, *Ae*. *fluviatilis* and *Ae*. *scapularis*, comprised 68% of all the mosquitoes collected in the study areas. Other species frequently found in the urban parks surveyed were *Cx*. *declarator*, *Ae*. *aegypti*, *Cx*. *chidesteri*, *Limatus durhami* and *Cx*. *lygrus*. The observed richness ranged from 16 species in Ibirapuera Park to 47 in Anhanguera Park (see Supplementary Table S2 for further information on mosquito richness, composition and abundance of larval and adult forms in each park).

The sample-based species accumulation curves (1,000 randomizations without replacement) showed a tendency to reach an asymptote after the study period (see Supplementary Figure S3). Observed richness values were within the estimated richness confidence interval for the nine areas surveyed (see Supplementary Figure S1). Rarefied species richness based on random resampling of 1,205 individuals (the number of mosquitoes collected in the Alfredo Volpi Park, which had the lowest abundance among the parks studied) confirmed that more species would be found in species-rich parks than in species-poor parks if the sampling effort was based on the same number of individuals (see Supplementary Figure S2). Species evenness based on Pielou’s index (J) ranged from 0.29 (Alfredo Volpi park) to 0.75 (Ibirapuera Park) for mean values and from 0.23 (Alfredo Volpi park) to 0.78 (Chico Mendes park) for median values (see Supplementary Figure S4). Exploratory analysis based on Spearman’s Rank-order correlation showed no evidence of an association between species richness and mean (rho = −0.483, *p* = 0.194) or median evenness (rho = −0.333, *p* = 0.385). The Sørensen similarity index ranged from 0.81, for Alfredo Volpi Park and Previdência Park, to 0.36, for Anhanguera Park and Ibirapuera Park (see Supplementary Table S3).

### Mosquito-richness predictive models

**Figure 2:**
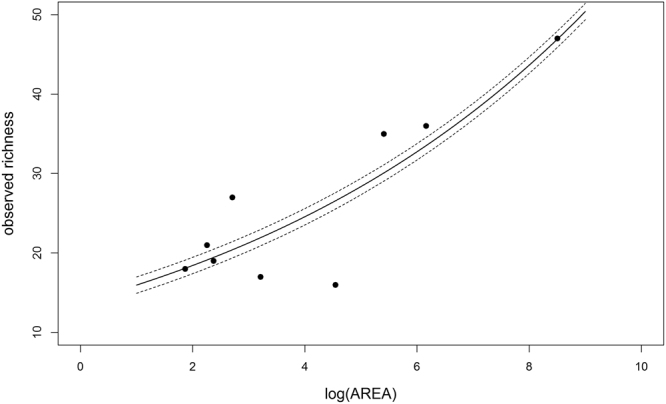
Species-area relationship for mosquito richness found in urban parks in the city of São Paulo, Brazil. The graph shows the observed richness (dots) and the predicted richness (continuous line) based on the linear model S = 2.626 + 0.144*log*AREA*, where 2.626 is the fitted intercept and 0.144 the fitted slope. The dashed lines represent the mean standard error of the estimated slope (0.028).

Model selection based on the Akaike information criterion corrected for small samples (AICc) suggests that the model predicting a species-area relationship, according to which richness is positively associated with the log of the area (logAREA), had the greatest support (∆AICc = 0). This model provided the highest strength of evidence (weight = 0.483) and explanatory power (pseudo-R² = 0.333) of all the models analyzed. The model that considers only the log proximity index, which combines patch isolation and surrounding fragmentation and is abbreviated here as log*PROX*, had less empirical support and explanatory power than the species-area model but predicted species richness more accurately than the model considering only the intercept (null model). The models considering both log*AREA* and log*PROX* (additive and interaction effects) had less support in the model selection. The null model showed insufficient strength of evidence (weight = <0.001) and a high AICc value compared with the other models (Table 1). Figure 2 shows the relationship between mosquito richness and log area according to the empirical data collected in our study.

**Table 1:** Candidate models for predicting mosquito richness and similarity in urban green spaces.

Response variable	Models	Intercept (*a*)	Slope	Phi	AICc	∆ AICc	Weight	Pseudo-R²
Species Richness (S)	***a*** + ***b*** *log ***AREA***	**2**.**626 (0**.**151)**	***b*** **=** **0**.**144 (0**.**028)**	**—**	**60**.**6**	**0**	**0**.**803**	**0**.**311**
	*a* + b*log*PROX*	2.496 (0.189)	*b* = 0.133 (0.029)	—	64.4	3.8	0.119	0.263
	*a* + b*log*AREA* + c*log*PROX*	2.659 (0.165)	*b* = 0.164 (0.083) *c* = −0.021 (0.082)	—	65.3	4.7	0.075	0.318
	*a* + b*log*AREA* + c*logPROX + d*logAREA*log*PROX*	2.896 (0.414)	*b* = 0.078 (0.156) *c* = −0.047 (0.090) *d* = 0.009 (0.015)	—	72.1	11.5	0.002	0.317
	*a*	3.266 (0.065)	—	—	81.7	21.2	<0.001	—
Species similarity (Ss)	***a*** **+** ***b*** * ***SA*** + ***c*** * ***SB*** + ***d*** * ***SA*** * ***SB***	**2**.**472 (0**.**389)**	***b*** **=** **−0**.**078 (0**.**012)** ***c*** **=** **−0**.**058 (0**.**019)** ***d*** **=** **0**.**002 (0**.**0006)**	**80**.**85 (18**.**95)**	**−97**.**2**	**0**	**0**.**991**	**0**.**797**
	*a* + b*SA + c*SB	1.078 (0.188)	*b* = −0.033 (0.004) *c* = 0.012 (0.008)	56.72 (13.26)	−87.1	10.1	0.006	0.586
	*a* + b*SA + c*SB + d*DIST	1.058 (0.186)	*b* = −0.034 (0.004) *c* = 0.010 (0.008) *d* = 0.005 (0.005)	58.55 (13.69)	−85.5	11.6	0.003	0.606
	*a* + b*DIST	0.432 (0.152)	*b* = −0.002 (0.007)	21.10 (4.86)	−56.4	40.8	<0.001	0.002
	*a*	0.391 (0.072)	—	21.04 (4.85)	−54.1	43.1	<0.001	—

For each model the intercept (a), slopes (b, c, d), dispersion parameter (phi - Φ), Akaike information criterion for small samples (AICc and ∆AICc), Akaike weight and Pseudo-R² are shown. The standard error of estimates is shown in brackets. The explanatory variables are patch size (*AREA* **)**, proximity index (*PROX*), species richness for sites A and B (*SA* and *SB*) and geographic distance between sites (*DIST*).

### Species-similarity predictive models

**Figure 3:**
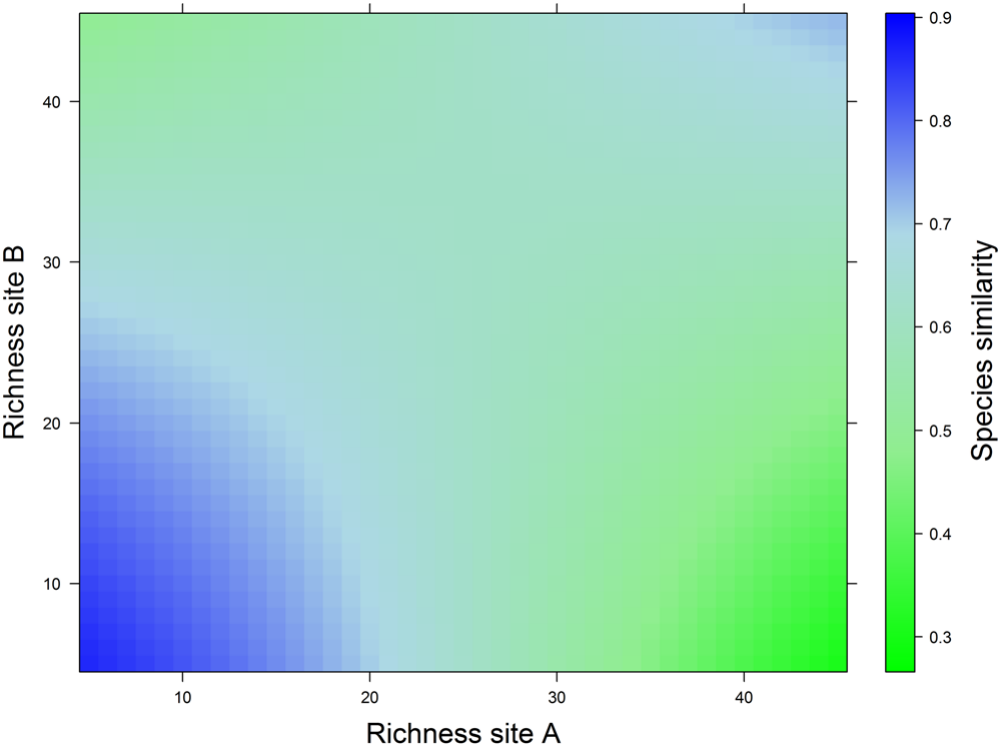
Predicted values for species similarity (Ss) based on the model Ss = 2.472 − 0.078*SA − 0.058*SB + 0.002*SA*SB, where *SA* and *SB* are the mosquito richness in two hypothetical sites A and B, respectively, 2.472 is the fitted intercept and −0.078, −0.058 and 0.002 are the fitted slopes. Species similarity is represented by different colors.

AICc model selection indicated that species similarity is best explained by the model that includes the species richness in site A (*SA*) and site B (*SB*) and the product of these variables (Table 1). This model had more strength of evidence (weight = 0.991) and better explanatory power (Pseudo-R² = 0.797) than the competing models, all of which had little empirical support and less explanatory power than the best model (Table 1). Figure 3, which is based on values predicted by the best model, shows how the similarity in mosquito composition tends to increase with a reduction in species richness at two hypothetical sites (A and B).

### Nestedness analysis

The observed nestedness based on overlap and decreasing fill (NODF) showed greater nestedness than expected by chance alone for both sites and species. Nestedness was determined using 1,000 null matrices generated by three different null model algorithms. Mosquito assemblages in species-poor parks tended to constitute subsets of progressively more species-rich ones (N rows), and less frequently observed mosquitoes tended to be found in subsets of the parks where the most widespread species were present (N columns) (Table 2).

**Table 2:** Probability (*p*) of the chance occurrence of the observed values of nestedness based on overlap and decreasing fill (NODF).

NODF statistic	Value	Null model algorithm		
		r00_both	swsh_both	r2dtable
N.columns	38.737	<0.001	<0.001	<0.001
N.rows	47.672	<0.001	0.008	<0.001
NODF	38.910	<0.001	<0.001	<0.001

Values determined using 1,000 random matrices generated by three quantitative null model algorithms.

### Vector mosquitoes and their nested order in the assemblages

**Figure 4:**
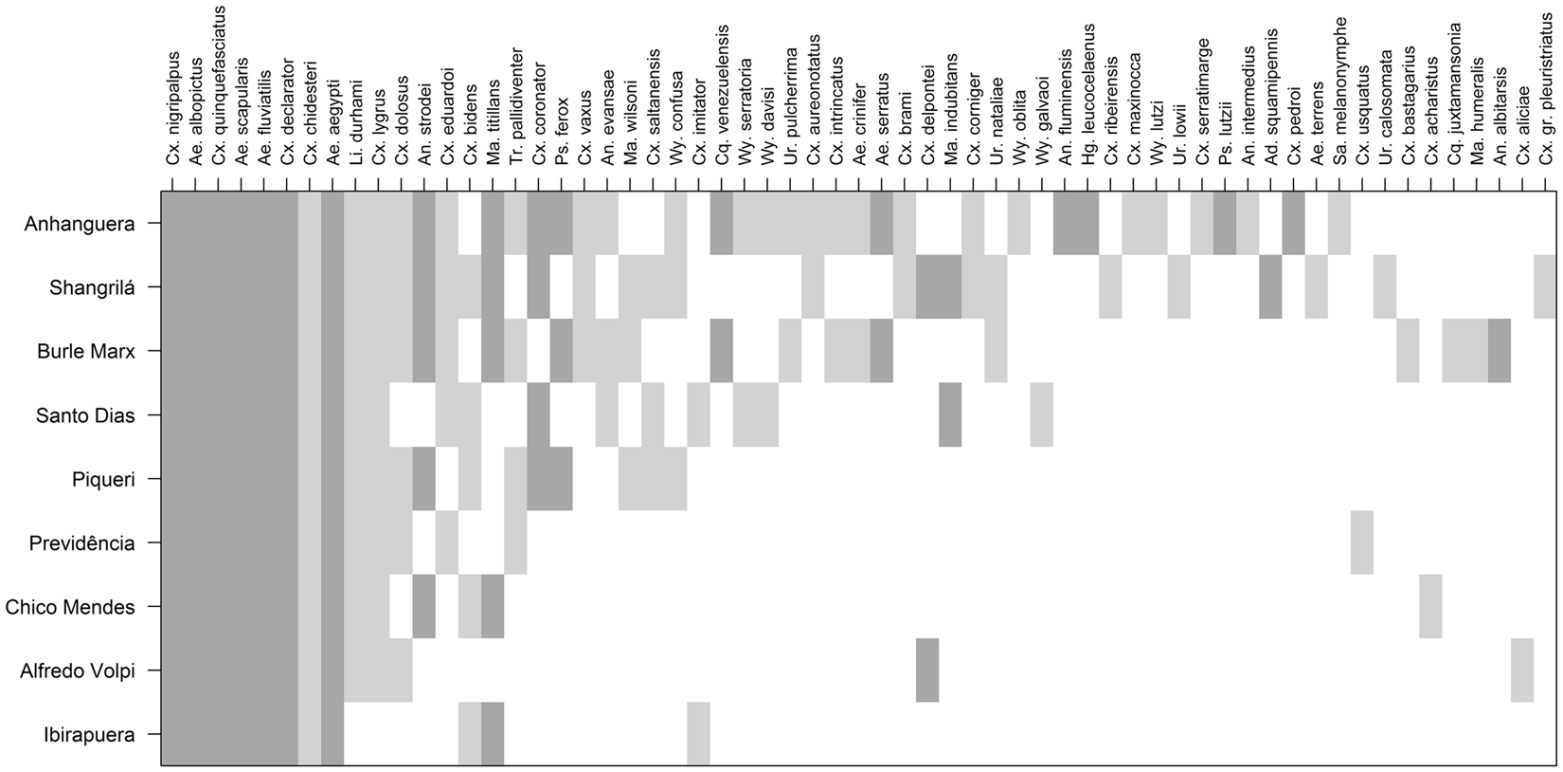
Matrix of data ordered by marginal totals for species richness (parks surveyed in rows) and total abundance (mosquito species in columns). Cells filled in light and dark gray indicate that the species was found in the site. Vector mosquitoes are represented by dark gray cells.

Among the species collected in our study, 24 have already been found carrying pathogens in natural habitats and/or their vector competence has been proven in experimental studies (see Supplementary Table S4 for species, pathogens and supporting references). To date, the species *Aedeomyia squamipennis*, *Culex chidesteri*, *Culex saltanensis* and *Trichoprosopon pallidiventer* have only been found carrying arboviruses and wild plasmodia that do not affect humans and were thus not considered vectors of human pathogens. Of the 20 species that were, the following seven were the most common and abundant in the study areas: *Cx*. *nigripalpus*, *Ae*. *albopictus*, *Cx*. *quinquefasciatus*, *Ae*. *scapularis*, *Ae*. *fluviatilis*, *Cx*. *declarator* and *Ae*. *aegypti*. Together these represent approximately 73% of all the mosquitoes collected in the study. Figure 4 shows a matrix of the data ordered by marginal totals for species richness (sites in rows) and abundance (species in columns). The cells on the left-hand side of the graph (more frequent and more abundant species) are dominated by vector mosquitoes.

## Discussion

We have reported evidence that mosquito richness and composition in urban parks can follow the patterns predicted by the ETIB, with a tendency toward a species-area relationship in which larger areas tend to be more species-rich than smaller ones and toward a nested pattern that increases similarity between assemblages in species-poor sites, indicating a non-random process of species loss or gain. Vector mosquitoes such as *Cx*. *nigripalpus*, *Ae*. *scapularis*, *Ae*. *fluviatilis* and *Cx*. *declarator* as well as the ‘urban exploiters’ *Ae*. *aegypti*, *Ae*. *albopictus* and *Cx*. *quinquefasciatus* were among the most common and abundant species in the urban green spaces in our study and were collected in both species-rich and species-poor locations. This may indicate that a progressive, selective species loss partly explained by a reduction in habitat size leads to increasing similarity in mosquito composition and a tendency for scenarios to develop in which mosquito assemblies in species-poor urban green spaces are composed largely of species considered vectors of human pathogens.

The association between the area available for habitation and species richness is one of the most fundamental relationships in ecology^20,31^. It has been used extensively in the literature to explain urban-insect species richness in urban green spaces^14,18^. Faeth and Kane^32^, studying Diptera and Coleoptera from nine urban parks in the city of Cincinnati, Ohio, USA, found a relationship between area and species richness and suggested that large green areas would tend to reduce the risk of extinction of specialized species. A species-area relationship was also found for dipterans in a study of 40 green spaces in California, USA^33^, for spiders and carabid beetles collected in urban green spaces in Tokyo and Yokohama, Japan^34,35^, for butterflies and carabid beetles in protected areas in the city of Halle, Germany^36^, and for hemipterans in 18 roundabouts in the city of Bracknell, England^37^.

There is a dearth of studies on the relationship between mosquito species richness and habitat size in urban areas. However, a study conducted by Chaves *et al*.^15^ surveyed eight sites in southwest Chicago, Illinois, USA, and found no association between habitat size and mosquito richness, but found an association between richness and landscape heterogeneity. As the authors found no relationship between landscape heterogeneity and habitat size, they suggested that richness may be more associated with the presence of different mosquito larval habitats than with habitat size. In fact, some studies of urban green spaces have shown that the species-area relationship tends to be explained partly by the fact that large areas have greater habitat diversity than smaller ones^38,39^. In addition, at patch level, species richness may also respond positively or negatively to factors such as the edge effect, the shape of the patch^40,41^, the age and history of the forest fragment studied and the surrounding land use^16,42^.

Another important factor that may influence species richness in urban forest fragments is the degree of isolation and fragmentation, which has been shown to be important when determining the richness of carabid beetle communities in different cities^43–45^. In the present study, habitat size was shown to be a better predictor of mosquito richness than the degree of patch isolation although when the latter was considered the only explanatory variable, the model showed significantly better predictive power than the null model. This suggests that even though patch isolation has less influence on mosquito richness than habitat size, this variable can predict species richness to some degree.

Our results showed a significant nested pattern among sites and species, suggesting that habitat and species features may be responsible for the patterns observed. Hence, in addition to the effects that can be attributed to habitat characteristics such as size and isolation, species-dependent traits, such as dispersal ability and adaptation to human-made environments, must also be considered possible contributing factors. Nestedness patterns have been previously identified in urban insect assemblages^45–49^. For example, Soga *et al*.^48^ surveyed 20 forest remnants in Tokyo, Japan, and found evidence that a selective local extinction could be responsible for the nesting pattern of butterfly assemblages. In turn, Lizée *et al*.^49^, researching butterfly communities in 15 urban parks in the city of Marseille, France, showed a significant relationship between nested pattern and isolation, suggesting that species’ dispersal capacity plays an important role in assemblage formation.

According to the ETIB and the metapopulation and metacommunity theories, several regional and local processes may be responsible for the formation of species-area relationships and nested-subset patterns in insular fragments^50,51^. Large areas can exhibit more habitat heterogeneity and thus harbor more species, including those with greater requirements (habitat specialists)^52,53^. Small fragments are more susceptible to environmental disturbances, which affect mainly the permanence of low-abundance species and species that are not resistant to environmental stress, leading to selection of more abundant and well adapted species^54^. Larger areas may harbor more species simply because they function as “targets” that tend to receive effectively larger number of species than smaller ones^31^. Likewise, regionally abundant species and those with more dispersal ability would be more likely to colonize more sites than those with low density and less dispersability^51^. Larger fragments may also support larger populations of all species, reducing the stochastic probability of local extinction of certain species^17^. The patterns observed here are certainly due to a combination of these local and regional processes. However, we believe that the mosquito richness and composition patterns found in this study may have been influenced more by deterministic than by stochastic processes, since the common and more abundant species (i.e., those found in both large and small urban green spaces) tended to be those with a greater dispersal ability and greater adaptability to breeding in different types of larval habitats^55^, whereas most of the other mosquito species collected were found in only one or two sites (rare species) mainly in the larger parks where there are suitable environmental conditions and larval habitats to maintain their populations^56^.

One possible consequence of non-random extinction and colonization in urban green spaces is biotic homogenization^57^, which occurs when species similarity across a given area increases over time because of species invasions and extinctions. In many cities around the world this can be caused by homogeneous physical characteristics in urban areas, resulting in the selection of similar species from the pool of regional native or invasive species and allowing ‘urban exploiters’ to find similar habitats^58^. In insect communities, evidence of biotic homogenization has been found in butterflies, carabid beetles, pollinator communities, true bugs and leafhoppers^59–62^. Our data suggest that at low regional richness levels there is a tendency toward biotic homogenization of mosquito species in urban green spaces. It should be emphasized that some of the most frequent and abundant mosquito species found in this study are known to be well adapted to man-made environments and are also found in abundance in other Brazilian cities or even in other cities around the world^55,63–65^.

Mosquitoes of epidemiological interest such as *Cx*. *quinquefasciatus*, *Ae*. *aegypti* and *Ae*. *albopictus* may benefit from a reduction in urban green spaces, as this reduces the numbers of predators and competitors. Furthermore, they are less dependent on these areas than other species as they can colonize a vast range of breeding sites near or inside human dwellings. Little is known about the influence of urban green spaces on the prevalence of diseases transmitted between urban mosquitoes and humans, although a study conducted in the city of São Paulo found evidence that the incidence of dengue fever is lower in areas with greater vegetation cover, which reduces the urban heat island effect^66^. In our study we observed that although *Ae*. *aegypti* can use breeding sites within the parks to develop, its abundance was often lower in these areas than that of *Ae*. *albopictus* and *Cx*. *quinquefasciatus*. The abundance of the main vector mosquitoes may respond differently to variations in the size and species richness of urban parks. For example, while *Ae*. *aegypti* appeared to be more abundant in smaller parks, the opposite appeared to be true for *Cx*. *nigripalpus*. We believe that an appropriate assessment of abundance patterns should take into account the different responses of each vector to seasonal climatic variations, as well as differences in the landscape and species diversity in each area.

One of the main limitations of the present study is the small number of parks surveyed, which could limit the strength of the conclusions and the extent to which they may be generalized. In addition, the study was not replicated in other cities. However, the fact that similar patterns to those observed here have also been found for other invertebrate taxa in urban green areas in other cities around the world lends weight to our findings. We believe that mosquitoes in urban green spaces are an appropriate subject for use with the ETIB because most mosquito species are found in natural or semi-natural areas. This was reflected in the high mosquito richness found in the urban parks surveyed. In addition to the species of urban mosquitoes that do not depend on green areas, we found at least seventy species that are to some extent dependent on these areas to develop and maintain their populations, i.e., many mosquitoes are restricted to these ‘green islands’.

A key point we observed is the tendency for species-poor mosquito assemblages to be composed largely of vectors of human pathogens. To better understand whether this pattern has any implications in the context of disease ecology, further studies will be needed. Firstly, it is necessary to know how often this pattern can be observed in large cities. Secondly, the role that urban green areas play in the maintenance or emergence and reemergence of vector-borne diseases is uncertain, particularly in relation to zoonotic pathogens that infect a wide variety of vector and host species (e.g., West Nile virus, Saint Louis encephalitis virus, yellow fever virus)^67^. For example, while the rate of interspecific encounters between highly competent vector mosquitoes and reservoir hosts may increase in species-poor urban green spaces^68^, increasing pathogen transmission and the risk of human infection, species loss in these areas may eliminate populations of mosquitoes responsible for maintaining enzootic cycles and even species that serve as a bridge for pathogen transmission between infected vertebrate hosts and humans.

Another important point is that not all the most common and abundant species of vectors found in the areas studied can be considered of public health concern, since only *Ae*. *aegypti*, *Ae*. *albopictus*, *Cx*. *nigripalpus* and *Cx*. *quinquefasciatus* have been shown to be important vectors of pathogens in urban areas and have been targets of mosquito control programs in many cities around the world^4,69,70^. As public health departments have limited resources, especially in less developed countries, it is unlikely that any efforts to monitor and control vector mosquitoes other than species considered the main vectors of diseases will be undertaken. Nevertheless, one must question whether mosquitoes that are considered less important vectors from the perspective of surveillance and control do indeed play an insignificant role or whether there is a lack of studies on the importance of these mosquitoes for human infection and pathogen maintenance in urban or peri-urban areas.

Since the human population living in urban areas tends to grow, an increase in the emergence and reemergence of vector-borne diseases can be expected in the coming decades^9,71^. Therefore, it is important that other studies be carried out to improve our understanding of how habitat loss and fragmentation due to urbanization affect the richness and composition of vector insects and how this may influence the risk of pathogen transmission. The use of predictive models based on ecological theories to explain the processes responsible for local and regional diversity patterns can provide valuable insights. Finally, it should be pointed out that the findings of this study may reflect the situation in other large urban centers because, as argued by McKinney^58^, cities are built to meet the demands of a single species, *Homo sapiens*, and are therefore physically very similar throughout the world.

## Materials and Methods

### Selection and characterization of the study areas

The city of São Paulo (23.54°W 46.63°S) is in southeastern Brazil. The climate is humid subtropical with mild, dry winters and rainy summers with moderately high temperatures^72^. The city extends over 1,521,110 square kilometers, of which approximately 970,000 is built up, and the population is currently estimated at 11.9 million, making it the largest and most populous city in the Americas and the southern hemisphere. It is also the main financial and commercial center in South America^73^. Before São Paulo was founded, the area it now occupies consisted basically of floodplains, fields and forests. The 1940s saw the start of an intense urbanization process, leading to rapid population growth and the consequent displacement of the population to outlying areas. These events in turn led to the gradual elimination of the native, natural vegetation. Most of the remaining green areas in the city are now urban parks and conservation units^74^.

From 59 candidate urban parks, in which preliminary assessments of mosquito fauna had been previously carried out^75^, nine were selected (Fig. 1): Alfredo Volpi Park (23°35.273′S 46°42.153′W), Anhanguera Park (23°25.208′S 46°46.626′W), Burle Marx Park (23°37.974′S 46°43.319′W), Chico Mendes Park (23°30.436′S 46°25.680′W), Ibirapuera Park (23°35.282′S 46°39.505′W), Piqueri Park (23°31.666′S 46°34.415′W), Previdência Park (23°34.851′S 46°43.633′W), Santo Dias Park (23°39.841′S 46°46.386′W) and Shangrilá Park (23°45.689′S 46°39.840′W. The parks were not selected at random as, based on our species-area-isolation hypothesis, fragments with different sizes and degrees of isolation should be selected (Supplementary Table S1). The number of sites used for the sample (nine) was limited because of the time and resources available. The distances between the parks varied from 2.1 to 38.9 km (see Supplementary Table S5). Monthly collections were performed in each park over one year. In Alfredo Volpi Park, Anhanguera Park, Chico Mendes Park, Ibirapuera Park, Santo Dias Park and Shangrilá Park, collections were made from March 2011 to February 2012, while in Burle Marx Park, Piqueri Park and Previdência Park they were performed from August 2012 to July 2013.

### Specimen Collection and identification

While some mosquito species are more easily collected in their adult form, others are only collected in their immature form. Therefore, for a comprehensive survey of species richness in a mosquito assemblage the use of different collection techniques is recommended. It is certainly more difficult to capture culicid diversity by larval sampling only. We therefore did not distinguish between larval and adult forms during the analyses as information on both forms is needed to ensure a realistic count of species number in the assemblages. Ideally, traps should be installed along transects or by random selection of subsample areas; however, we opted to set the traps in places where mosquitoes would most likely be encountered based on a knowledge of the bioecology of Culicidae insects.

Adult mosquitoes were captured with a battery-powered aspirator, CDC light traps and Shannon traps^76^. Aspirations were performed during the day in three fixed areas in each park for 20 minutes each. One of the aspirations was always performed near the administrative areas in the parks, where there are normally many staff and visitors, and the remaining two were performed in more natural locations near potential breeding sites. Four CO_2_-baited CDC light traps were set up in each park. Two locations were chosen in each park: one consisting of open, more urbanized areas with groves and grass (usually close to recreational and administrative areas), and the other in wooded areas less accessible to visitors. Two of the traps were placed at each location, one 1 m above the ground and another 8–10 m above the ground in the tree canopy to capture mosquitoes from different vegetation strata. The traps were left out for three hours beginning one hour before twilight. A Shannon light trap was installed in densely vegetated areas and the mosquitoes caught in the trap were collected on its surface with a manual electric aspirator over two hours starting at twilight.

Immature mosquitoes were collected with 400 mL larval dippers in natural larval habitats, such as puddles and ponds, or artificial ones, such as water reservoirs and containers. Suction samplers were used to collect samples from bromeliad axils, tree holes and bamboo internodes. The mosquito larvae collected in this way were kept in the laboratory until they reached adult stage.

Sampling effort was approximately 240 collection-hours in each park broken down as follows: 12 hours of aspiration, 24 hours of Shannon trap, 144 hours of CDC traps and 60 hours of active search for immature forms. Since the sampling effort in each study area was the same and was based on trap exposure time and an active search for immature forms, larger parks had proportionally less area sampled than smaller parks. Therefore, the possibility of underestimating the number of species increased with the size of the park.

Specimen identification was conducted at the Entomology Laboratory, School of Public Health, University of São Paulo (LESP/FSP/USP), and the Laboratory for Research into and Identification of Synanthropic Fauna at the Zoonosis Control Center in São Paulo (Labfauna/CCZ). Species identification was performed using morphological taxonomic keys^55,77–79^.

### Landscape metrics

Orbital images of the metropolitan region of São Paulo recorded on April 21, 2011, by the Landsat 5 satellite (TM sensor, bands 3 and 4) with a spatial resolution of 30 m were obtained from the Brazilian National Institute for Space Research (INPE) database. Images of red and near-infrared spectral bands were combined with QGIS^80^ and used to classify the landscape. Field observations and other satellite images were used to validate our landscape classification. The patches were classified as: (1) vegetation (forest, shrubs and undergrowth), (2) water bodies (rivers, ponds and streams) or (3) urban (bare ground, paved surfaces, roads and built-up areas). One-kilometer buffers around the parks were considered for the calculations, and the resulting mosaic was used to determine landscape metrics with Fragstats version 4.2^81^. The two patch-based landscape metrics used were (see values in Supplementary Table S1): (I) *Patch area* - the green area of each park measured in hectares. The total area covered by vegetation in the fragments was used to calculate the area of each park even when the patch extended beyond the administrative boundaries of the park; (II) *Proximity index* - measures the degree of patch isolation and fragmentation within the specified search radius and therefore takes into account the size and proximity of all the patches surrounding the focal patch^82^. To facilitate use of these metrics we performed a log transformation on both variables.

### Data analysis

Sample sufficiency was evaluated by plotting sample-based species accumulation curves, and total richness was estimated by the Chao 1 method (Chao, 1984) with 1000 randomizations without replacement and a 95% confidence interval. To compare species richness for a hypothetical sampling effort based on collection of the same number of individuals in each park, the rarefied species richness was evaluated by a random resampling of individuals based on the number of mosquitoes collected in the park with the lowest abundance (see Supplementary Figs S1, S2, and S3).

Although the sampling in the nine parks was performed in different years (six parks in 2011–2012 and three in 2012–2013), the effect of time was not considered because we assumed that the short period between the two sampling periods was not sufficient to produce a great variation in species richness or composition. For the analysis, we considered as diversity parameter a simple count of the number of species (richness), since this metric proved to be less biased than evenness and satisfied the hypothesis we were testing (species-area-isolation relationship). The evenness of mosquito assemblages is subject to variations arising from the life stage considered (larval forms, for example, have a more aggregated distribution than adults) and the collection techniques used (some species can be more easily detected by a specific trap or technique) and can show a temporal dynamic that is a function of climatic variables. For this reason, we opted not to evaluate evenness as a function of the size or isolation of the urban green areas. Despite these issues, we have included a boxplot of the variations in mosquito assemblage evenness throughout the study period for the nine parks (Supplementary Fig. S4). Pielou’s evenness index (J) was used for this, and only the monthly variation for adult forms was considered. An exploratory analysis based on Spearman’s rank-order correlation was also carried out to search for a possible correlation between species richness and evenness.

To test the applicability of the ETIB we evaluated the effects of patch size (*AREA*) and proximity index (*PROX*) on species richness (S). Five candidate statistical models were tested:

(I) S = *a* + b*log*AREA* − semi-log species-area variant of the Arrhenius^20^ power function (*S* = CA^z^);
(II) S = *a* + b*log*PROX*;
(III) S = *a* + b*log*AREA* + c*log*PROX*;
(IV) S = *a* + b*log*AREA* + c*log*PROX* + d*logAREA*log*PROX*;
(V) S = *a* (null model),

where *a* is the fitted intercept and *b*, *c*, and *d* are the fitted slopes.

A second set of models was proposed to test the hypothesis that species loss tends to increase species similarity in mosquito assemblages in urban green areas because species adapted to human-altered environments tend to be selected throughout the urbanization process^58^. We also considered the effect of the distance in kilometers between the fragments on species similarity to test for a possible species turnover with distance between the sites studied. Therefore, species similarity (Ssim) calculated by the Sørensen similarity index^83^ was considered the response variable. The observed richness *SA* and *SB* (the species richness observed at sites A and B in the pairwise comparison of parks) and the geographic distance between the studied areas (*Dist*) were considered predictor variables. The models make the following predictions:

(I) Ssim = *a* + b*SA + c*SB;
(II) Ssim = *a* + b*SA + c*SB + d*SA*SB;
(III) Ssim = *a* + b*Dist;
(IV) Ssim = *a* + b*SA + c*SB + d*Dist;
(V) Ssim = *a*,

where *a*, *b*, c, and *d* are the fitted model parameters as described above.

For statistical modeling we used the generalized linear model (GLM) with Poisson errors (log link function) for the species-richness models and beta regression with beta errors (logit link function) for the species-similarity models^84^. The parameters were estimated by the maximum likelihood method and an information-theoretical approach based on the Akaike information criterion corrected for small samples (AICc) was applied to select the most plausible statistical models^85^. The fitted models were tested for variable collinearity, and the models with Poisson errors were also tested for under/overdispersal. As none of them had dispersal parameters statistically different from 1, it was not considered necessary to use a quasi-Poisson or negative-binomial model to correct standard errors. The models with the smallest AICc were considered the best, and ∆AICc ≤2 was adopted as the cutoff to select models with more empirical support. The strength of evidence in favor of each model was evaluated using Akaike weights^85^. Explanatory power was determined using McFadden’s pseudo-R², for which larger values suggest a better fit^86^.

To evaluate whether mosquito assemblages found in species-poor sites are subsets of assemblages found in species-rich sites we performed a nestedness analysis^87^. First, we ordered a matrix by marginal totals for species richness (sites in rows) and abundance (species in columns) and calculated the nestedness based on overlap and decreasing fill (NODF). This can measure independently whether species-poor sites constitute subsets of progressively richer ones (N rows) and whether the less frequent species are found in subsets of sites where the most widespread ones occur (N columns). It also provides a measure of nesting for the whole matrix^88^. To test the non-randomness of the results, 1,000 random matrices were generated using three different quantitative null model algorithms: *r00_both*, which first randomizes the presence data in the matrix (i.e., non-zero cells) and then shuffles individuals over non-zero cells so that only the total sum of the matrix remains constant; *swsh_both*, which fills the matrix randomly, preserving row and column frequencies, and shuffles individuals over non-zero cells; and *r2dtable*, which fills the matrix randomly, preserving row and column sums of individuals but not marginal frequencies^89^. All analyses were carried out with R version 3.1.1^90^.

Finally, an extensive literature and database search was conducted to determine which of the species in our study have already been found in the natural environment to carry mosquito-borne pathogens that cause diseases in humans or have had their vector competence for these pathogens proven in experimental studies (see Supplementary Table S4). This information was compared with the study findings to identify which of these species tend to persist and thrive when biodiversity is lost in urban green areas.

### Data availability

All data generated or analysed during this study are included in this published article (and its Supplementary Information files).

### Ethics statement

The study was approved by the Ethical Committee of the University of São Paulo (FSP/USP—Project 000304), and collection permits were provided by the São Paulo Department of Parks and Green Spaces (Permit 345/2010).

## Electronic supplementary material

Supplementary Information
