# Supplementary material for: Mosquitoes in urban green spaces: using an island biogeographic approach to identify drivers of species richness and composition
Source: Sci Rep. 2017 Dec 19;7:17826. doi: 10.1038/s41598-017-18208-x (PMC5736758; doi:10.1038/s41598-017-18208-x)
Supplement: Supplementary file 1 — Supplementary Information [file 41598_2017_18208_MOESM1_ESM.doc]

**Mosquitoes in urban green spaces: using an island biogeographic approach to identify drivers of species richness and composition**

**Antônio Ralph Medeiros-Sousa^1^, Aristides Fernandes^1^, Walter Ceretti-Junior^1^, André Barreto Bruno Wilke^1^ & Mauro Toledo Marrelli^1^**

**^1^Department of Epidemiology, School of Public Health, São Paulo University, Avenida Doutor**

**Arnaldo 715, CEP 01246-904, São Paulo, Brazil.**

**Supplementary Information**

**Table S1.** Species richness, log of the area (log AREA) and log of the proximity index (log PROX + 1) for green fragments in nine urban parks in the city of São Paulo, Brazil.

| **Park** | **Species richness** | **log AREA** | **log PROX + 1** |
| --- | --- | --- | --- |
| Alfredo Volpi | 17 | 3.212455 | 4.7433061 |
| Anhanguera | 47 | 8.502806 | 8.6478821 |
| Burle Marx | 35 | 5.408068 | 6.390079 |
| Chico Mendes | 18 | 1.868721 | 2.660169 |
| Ibirapuera | 16 | 4.544252 | 4.3937262 |
| Piqueri | 21 | 2.255493 | 0.7317283 |
| Previdência | 19 | 2.371178 | 3.6394358 |
| Santo Dias | 27 | 2.710048 | 3.0385414 |
| Shangrilá | 36 | 6.160131 | 5.8858828 |

**Table S2**. Number of adults (A) and immature (I) specimens by taxon/species collected in urban parks in the city of São Paulo, Brazil. Twelve collections were performed in each park from March 2011 to February 2012 (Alfredo Volpi Park, Anhanguera Park, Chico Mendes Park, Ibirapuera Park, Santo Dias Park and Shangrilá Park) and August 2012 to July 2013 (Burle Marx Park, Piqueri Park and Previdência Park).

| **Species** | **Parks** | | | | | | | | | | | | | | | | | | |
| --- | --- | --- | --- | --- | --- | --- | --- | --- | --- | --- | --- | --- | --- | --- | --- | --- | --- | --- | --- |
|  | **Alfredo Volpi** | | **Anhanguera** | | **Burle Marx** | | **Chico Mendes** | | **Ibirapuera** | | **Piqueri** | | **Previdência** | | **Santo Dias** | | **Shangrilá** | | **Total** |
|  | **A** | **I** | **A** | **I** | **A** | **I** | **A** | **I** | **A** | **I** | **A** | **I** | **A** | **I** | **A** | **I** | **A** | **I** |  |
| *Aedeomyia* (*Aedeomyia*) *squamipennis* (Lynch Arribálzaga, 1878) |  |  |  |  |  |  |  |  |  |  |  |  |  |  |  |  | 2 |  | 2 |
| *Aedes* (*Ochlerotatus*) *crinifer* (Theobald, 1903) |  |  | 4 |  | 11 |  |  |  |  |  |  |  |  |  |  |  |  |  | 15 |
| *Aedes* (*Ochlerotatus*) *fluviatilis* (Lutz, 1904*)* | 1006 |  | 1 | 17 | 1264 |  | 151 |  | 235 | 12 | 1104 |  | 157 |  | 231 |  | 150 | 14 | 4342 |
| *Aedes* (*Ochlerotatus*) *scapularis* (Rondani, 1848) | 39 |  | 100 | 1 | 1241 |  | 1110 |  | 25 | 31 | 1095 |  | 484 |  | 69 |  | 42 |  | 4237 |
| *Aedes* (*Ochlerotatus*) *serratus* (Theobald, 1901) |  |  | 2 |  | 3 |  |  |  |  |  |  |  |  |  |  |  |  |  | 5 |
| *Aedes* (*Protomacleaya*) *terrens* (Walker, 1856) |  |  |  |  |  |  |  |  |  |  |  |  |  |  |  |  | 1 |  | 1 |
| *Aedes* (*Stegomyia*) *aegypti* (Linnaeus, 1762) | 1 |  |  | 2 |  | 1 | 3 | 11 |  | 14 | 13 | 404 | 21 | 77 | 1 | 13 | 2 |  | 563 |
| *Aedes* (*Stegomyia) albopictus* (Skuse, 1895) | 5 |  | 5 | 359 | 12 | 79 | 15 | 52 | 20 | 613 | 55 | 3520 | 69 | 437 | 13 | 51 | 118 | 568 | 5991 |
| *Anopheles* (*Anopheles*) *fluminensis* Root, 1927 |  |  | 3 | 11 |  |  |  |  |  |  |  |  |  |  |  |  |  |  | 14 |
| *Anopheles* (*Anopheles*) *intermedius* (Peryassú, 1908) |  |  |  | 3 |  |  |  |  |  |  |  |  |  |  |  |  |  |  | 3 |
| *Anopheles* (*Nyssorhynchus*) *evansae* (Brèthes, 1926) |  |  | 2 | 8 | 9 | 14 |  |  |  |  |  |  |  |  | 1 |  |  |  | 34 |
| *Anopheles* (*Nyssorhynchus*) *strodei* Root, 1926 |  |  | 2 | 4 | 55 | 58 | 1 |  |  |  | 1 | 1 |  |  |  |  | 1 |  | 123 |
| *Anopheles* (*Nyssorhynchus*) *albitarsis* Lynch Arribálzaga, 1878 |  |  |  |  | 1 |  |  |  |  |  |  |  |  |  |  |  |  |  | 1 |
| *Coquillettidia* (*Rhynchotaenia*) *chrysonotum/albifera* |  |  | 1 |  | 1 |  |  |  |  |  |  |  |  |  |  |  |  |  | 2 |
| *Coquillettidia* (*Rhynchotaenia*) *venezuelensis* (Theobald, 1912) |  |  | 1 |  | 1 |  |  |  |  |  |  |  |  |  |  |  |  |  | 2 |
| *Coquillettidia* (*Rhynchotaenia*) *juxtamansonia* (Chagas, 1907) |  |  |  |  | 1 |  |  |  |  |  |  |  |  |  |  |  |  |  | 1 |
| *Culex* (*Culex*) *acharistus* Root, 1927 |  |  |  |  |  |  | 1 |  |  |  |  |  |  |  |  |  |  |  | 1 |
| *Culex* (*Culex*) *bidens* Dyar, 1922 |  |  |  |  |  |  | 2 |  | 4 |  | 2 |  |  |  | 18 |  | 169 |  | 195 |
| *Culex* (*Culex*) *brami* Forattini, Rabello & Lopes, 1967 |  |  |  | 12 |  |  |  |  |  |  |  |  |  |  |  |  | 1 |  | 13 |
| *Culex* (*Culex*) *chidesteri* Dyar, 1921 | 5 |  | 15 |  | 12 | 15 | 181 |  | 10 | 2 | 37 |  | 4 |  | 105 |  | 708 | 32 | 1126 |
| *Culex* (*Culex*) *coronator* Dyar & Knab, 1906 |  |  | 1 |  |  |  |  |  |  |  | 1 |  |  |  | 1 |  | 1 |  | 4 |
| *Culex* (*Culex*) *declarator* Dyar & Knab, 1906 | 3 |  | 14 |  | 13 |  | 338 |  | 29 |  | 204 |  | 44 | 31 | 56 |  | 489 | 3 | 1224 |
| *Culex* (*Culex*) *dolosus* (Lynch Arribálzaga, 1891) |  | 2 |  | 14 | 3 | 126 |  |  |  |  |  | 2 | 1 | 12 |  |  | 1 |  | 161 |
| *Culex* (*Culex*) *dolosus/eduardoi* |  |  | 1 |  | 5 |  | 12 |  | 3 |  | 20 |  | 1 |  | 2 |  | 23 |  | 67 |
| *Culex* (*Culex*) *eduardoi* Casal & García, 1968 |  |  |  | 84 | 3 |  |  |  |  |  |  |  |  | 10 |  | 2 |  | 63 | 162 |
| *Culex* (*Culex*) gr. Coronator |  |  | 4 | 86 |  |  |  |  | 3 |  |  |  |  |  | 7 |  | 3 | 36 | 139 |
| *Culex* (*Culex*) *lygrus* Root, 1927 | 1 |  | 18 |  | 3 |  | 9 |  |  |  | 13 |  | 7 |  | 8 |  | 10 |  | 69 |
| *Culex* (*Culex*) *nigripalpus* Theobald, 1901 | 1 |  | 2519 | 3 | 790 |  | 12 |  | 51 |  | 721 |  | 197 |  | 538 |  | 1320 | 31 | 6183 |
| *Culex* (*Culex*) *quinquefasciatus* Say, 1823 | 16 | 42 | 456 | 537 | 141 |  | 21 |  | 72 | 1316 | 239 | 1131 | 57 | 268 | 46 | 22 | 105 | 599 | 5068 |
| *Culex* (*Culex*) *saltanensis* Dyar, 1928 |  |  |  |  |  |  |  |  |  |  | 3 |  |  |  | 1 |  | 15 |  | 19 |
| *Culex (Culex*) spp. | 63 |  | 414 |  | 144 |  | 2504 |  | 110 |  | 499 |  | 108 |  | 459 |  | 1591 |  | 5892 |
| *Culex* (*Culex*) *usquatus* Dyar, 1918 |  |  |  |  |  |  |  |  |  |  |  |  | 1 |  |  |  |  |  | 1 |
| *Culex* (*Melanoconion*) *aliciae* Duret, 1953 | 1 |  |  |  |  |  |  |  |  |  |  |  |  |  |  |  |  |  | 1 |
| *Culex* (*Melanoconion*) *aureonotatus* Duret & Barreto, 1956 |  |  |  | 1 |  |  |  |  |  |  |  |  |  |  |  |  | 58 |  | 59 |
| *Culex* (*Melanoconion*) *bastagarius* Dyar & Knab, 1906 |  |  |  |  | 1 |  |  |  |  |  |  |  |  |  |  |  |  |  | 1 |
| *Culex* (*Melanoconion*) *delpontei* Duret, 1969 | 1 |  |  |  |  |  |  |  |  |  |  |  |  |  |  |  | 3 |  | 4 |
| *Culex* (*Melanoconion*) *intrincatus* Brèthes, 1916 |  |  | 1 | 22 |  | 9 |  |  |  |  |  |  |  |  |  |  |  |  | 32 |
| *Culex* (*Melanoconion*) cf. *maxinocca* Dyar, 1920 |  |  | 3 | 23 |  |  |  |  |  |  |  |  |  |  |  |  |  |  | 26 |
| *Culex* (*Melanoconion*) *pedroi* Sirivanakarn & Belkin, 1980 |  |  |  | 2 |  |  |  |  |  |  |  |  |  |  |  |  |  |  | 2 |
| *Culex* (*Melanoconion*) *ribeirensis* Forattini & Sallum, 1985 |  |  |  |  |  |  |  |  |  |  |  |  |  |  |  |  | 18 |  | 18 |
| *Culex* (*Melanoconion*) sec. Melanoconion |  |  | 2 | 31 | 26 | 15 |  |  |  |  |  |  |  |  | 1 |  | 1 |  | 76 |
| *Culex* (*Melanoconion*) *serratimarge* Root, 1927 |  |  | 4 | 3 |  |  |  |  |  |  |  |  |  |  |  |  |  |  | 7 |
| *Culex* (*Melanoconion*) spp. | 7 | 2 |  |  | 3 | 2 | 1 |  |  |  |  |  |  |  |  |  |  |  | 15 |
| *Culex* (*Melanoconion*) *vaxus* Dyar, 1920 |  |  | 2 | 3 | 8 | 14 |  |  |  |  |  |  |  |  |  |  | 51 |  | 78 |
| *Culex* (*Microculex*) gr. Imitator |  |  |  |  |  |  |  |  |  |  |  |  |  | 2 | 11 |  |  |  | 13 |
| *Culex* (*Microculex*) gr. Pleuristriatus |  |  |  |  |  |  |  |  |  |  |  |  |  |  |  |  |  | 1 | 1 |
| *Culex* (*Microculex*) *imitator imitator* Theobald, 1903 |  |  |  |  |  |  |  |  |  | 4 |  |  |  |  |  | 443 |  |  | 447 |
| *Culex* (*Phenacomyia*) *corniger* Theobald, 1903 |  |  | 1 |  |  |  |  |  |  |  |  |  |  |  |  |  | 2 |  | 3 |
| *Haemagogus* (*Conopostegus*) *leucocelaenus* (Dyar & Shannon, 1924) |  |  |  | 5 |  |  |  |  |  |  |  |  |  |  |  |  |  |  | 5 |
| *Limatus durhami* Theobald, 1901 | 1 |  | 1 | 142 | 23 | 263 |  | 8 |  |  | 1 | 7 | 5 | 110 | 3 | 7 | 5 | 305 | 881 |
| *Mansonia* (*Mansonia*) *humeralis* Dyar & Knab, 1916 |  |  |  |  | 1 |  |  |  |  |  |  |  |  |  |  |  |  |  | 1 |
| *Mansonia* (*Mansonia*) *indubitans* Dyar & Shannon, 1925 |  |  |  |  |  |  |  |  |  |  |  |  |  |  | 1 |  | 2 |  | 3 |
| *Mansonia* (*Mansonia*) *titillans* (Walker, 1848) |  |  | 2 |  | 1 |  | 54 |  | 1 |  |  |  |  |  |  |  | 15 |  | 73 |
| *Mansonia* (*Mansonia*) *wilsoni* (Barreto & Coutinho, 1944) |  |  |  |  | 1 |  |  |  |  |  | 1 |  |  |  |  |  | 31 |  | 33 |
| *Psorophora* (*Janthinosoma*) *ferox* (von Humboldt, 1819) |  |  | 46 |  | 1 |  |  |  |  |  | 6 |  |  |  |  |  |  |  | 53 |
| *Psorophora* (*Janthinosoma*) *lutzii* (Theobald, 1901) |  |  |  | 3 |  |  |  |  |  |  |  |  |  |  |  |  |  |  | 3 |
| *Sabethes* (*Sabethinus*) *melanonymphe* Dyar, 1924 |  |  | 1 |  |  |  |  |  |  |  |  |  |  |  |  |  |  |  | 1 |
| *Trichoprosopon* (*Trichoprosopon*) *pallidiventer/castroi/simile* |  |  |  |  |  |  |  |  |  |  |  |  |  | 1 |  |  |  |  | 1 |
| *Trichoprosopon* (*Trichoprosopon*) *pallidiventer* (Lutz, 1905) |  |  |  | 5 |  | 1 |  |  |  |  |  | 2 |  |  |  |  |  |  | 8 |
| *Trichoprosopon* spp. | 1 |  |  |  |  |  | 1 |  |  |  |  |  |  |  |  |  |  |  | 2 |
| *Toxorhynchites* spp. |  | 8 |  | 23 | 2 | 38 |  |  |  | 26 |  |  | 1 | 11 |  | 7 |  | 10 | 126 |
| *Uranotaenia* (*Uranotaenia*) *calosomata* Dyar & Knab, 1907 |  |  |  |  |  |  |  |  |  |  |  |  |  |  |  |  | 1 |  | 1 |
| *Uranotaenia* (*Uranotaenia*) *lowii* Theobald, 1901 |  |  |  |  |  |  |  |  |  |  |  |  |  |  |  |  | 6 |  | 6 |
| *Uranotaenia (Uranotaenia) nataliae* Lynch Arribálzaga, 1891 |  |  |  |  |  | 1 |  |  |  |  |  |  |  |  |  |  |  | 1 | 2 |
| *Uranotaenia* (*Uranotaenia*) *pulcherrima* Lynch Arribálzaga, 1891 |  |  |  | 3 |  | 2 |  |  |  |  |  |  |  |  |  |  |  |  | 5 |
| *Wyeomyia* (*Miamyia*) *lutzi* (da Costa Lima, 1930) |  |  |  | 7 |  |  |  |  |  |  |  |  |  |  |  |  |  |  | 7 |
| *Wyeomyia* (*Miamyia*) *oblita* (Lutz, 1904) |  |  |  | 110 |  |  |  |  |  |  |  |  |  |  |  |  |  |  | 110 |
| *Wyeomyia* (*Phoniomyia*) *davisi* (Lutz, 1904) |  |  |  | 13 |  |  |  |  |  |  |  |  |  |  | 1 | 59 |  |  | 73 |
| *Wyeomyia* (*Phoniomyia*) cf. *galvaoi* (Correa & Ramalho, 1956) |  |  |  |  |  |  |  |  |  |  |  |  |  |  | 3 | 53 |  |  | 56 |
| *Wyeomyia* (*Phoniomyia*) sp. |  |  |  |  |  |  |  |  |  |  |  |  |  |  | 1 |  |  |  | 1 |
| *Wyeomyia* (*Prosopolepis*) *confusa* (Lutz, 1905) |  |  | 1 |  |  |  |  |  |  |  | 1 |  |  |  |  |  | 7 |  | 9 |
| *Wyeomyia* (*Spilonympha*) *airosai/ howardi/*(*Dendromyia*) *luteoventralis* |  |  | 2 |  |  |  |  |  |  |  |  |  | 3 |  |  |  |  |  | 5 |
| *Wyeomyia serratoria* (Dyar & Nunez Tovar, 1927) |  |  |  | 49 |  |  |  |  |  |  |  |  |  |  |  | 1 |  |  | 50 |
| *Wyeomyia* spp. |  |  | 3 |  |  | 6 |  |  |  | 1 |  |  |  | 4 |  |  |  |  | 14 |
| **Total no. of specimens** | 1151 | 54 | 3632 | 1586 | 3780 | 644 | 4416 | 71 | 563 | 2019 | 4016 | 5067 | 1160 | 963 | 1577 | 658 | 4952 | 1663 | 37972 |
| **No. of observed species/taxa** | 15 | 4 | 32 | 31 | 29 | 16 | 17 | 3 | 12 | 9 | 19 | 7 | 16 | 11 | 23 | 10 | 33 | 12 | 73 |
| **Total no. observed species/taxa** | 17 | | 47 | | 35 | | 18 | | 16 | | 21 | | 19 | | 27 | | 36 | |  |

**Table S3.** Sørensen similarity index for pairwise comparisons of mosquito composition in urban parks in the city of São Paulo, Brazil.

|  | Anhanguera | Piqueri | Shangrilá | Burle Marx | Ibirapuera | Previdência | Chico Mendes | Santo Dias |
| --- | --- | --- | --- | --- | --- | --- | --- | --- |
| Piqueri | 0.55 |  |  |  |  |  |  |  |
| Shangrilá | 0.56 | 0.67 |  |  |  |  |  |  |
| Burle Marx | 0.65 | 0.63 | 0.56 |  |  |  |  |  |
| Ibirapuera | 0.36 | 0.60 | 0.47 | 0.45 |  |  |  |  |
| Previdência | 0.49 | 0.73 | 0.52 | 0.60 | 0.64 |  |  |  |
| Chico Mendes | 0.45 | 0.73 | 0.57 | 0.56 | 0.80 | 0.71 |  |  |
| Santo Dias | 0.51 | 0.67 | 0.58 | 0.49 | 0.65 | 0.65 | 0.65 |  |
| Alfredo Volpi | 0.42 | 0.69 | 0.53 | 0.52 | 0.67 | 0.81 | 0.74 | 0.61 |

**Table S4. Species collected in the study that have been found carrying pathogens in natural habitats or whose vector competence has been proven in experimental studies, pathogens carried by these vectors and the supporting references.**

| **Species** | **Pathogens** | **References** |
| --- | --- | --- |
| *Aedeomyia* (*Aedeomyia*) *squamipennis* (Lynch Arribálzaga, 1878)*** | Gamboa vírus | 91 |
| *Aedes* (*Ochlerotatus) fluviatilis* (Lutz, 1904) ** | yellow fever virus, *Plasmodium* gallinaceum, Dirofilaria immitis, dengue virus | 55,92-94 |
| *Aedes* (*Ochlerotatus*) *scapularis* (Rondani, 1848) | Melão virus, Ilhéus virus, Venezuelan equine encephalitis virus, Rocio virus, *Wuchereria bancrofti*, *Dirofilaria Immitis* | 95-98 |
| *Aedes* (*Ochlerotatus*) *serratus* (Theobald, 1901) | yellow fever virus, trocara virus; oropouche virus, Aura virus, Ilhéus vírus | 98,99 |
| *Aedes* (*Stegomyia*) *aegypti* (Linnaeus, 1762) * | dengue virus, yellow fever virus, Zika virus, Chikungunyia virus. | 98,100,101 |
| *Aedes* (*Stegomyia*) *albopictus* (Skuse, 1895)* | dengue virus, yellow fever virus, Zika virus, chikungunyia virus, Eastern equine encephalitis virus, La Crosse virus, Venezuelan equine encephalitis virus, West Nile virus, Japanese encephalitis virus among others. | 98,100-103 |
| *Anopheles* (*Anopheles*) *fluminensis* Root, 1927 | *Plasmodium malariae* | 104 |
| *Anopheles* (*Nyssorhynchus*) *strodei* Root, 1926 | *Plasmodium vivax; Plasmodium malariae* | 105,106 |
| *Anopheles* (*Nyssorhynchus*) *albitarsis* Lynch Arribálzaga, 1878 | *Plasmodium vivax, Plasmodium falciparum* | 98,105 |
| *Coquillettidia* (*Rhynchotaenia*) *venezuelensis* (Theobald, 1912) | Mayaro virus, Oropouche virus, Saint Louis virus | 107 |
| *Culex* (*Culex*) *chidesteri* Dyar, 1921*** | Nhumirim virus | 108 |
| *Culex* (*Culex*) *coronator* Dyar & Knab, 1906 | Saint Louis encephalitis virus, Mucambo virus, Eastern equine encephalitis virus, West Nile virus | 109-111 |
| *Culex* (*Culex*) *declarator* Dyar & Knab, 1906 | Saint Louis encephalitis virus | 109,112 |
| *Culex* (*Culex*) *nigripalpus* Theobald, 1901* | Saint Louis encephalitis virus, Eastern equine encephalitis virus, West Nile virus | 98,111,113,114 |
| *Culex* (*Culex*) *quinquefasciatus* Say, 1823* | *Wuchereria bancrofti*; Saint Louis encephalitis virus; Western equine encephalitis virus; West Nile virus, Japanese encephalitis virus, and others. | 95,111,114,116 |
| *Culex* (*Culex*) *saltanensis* Dyar, 1928*** | *Plasmodium juxtanucleare* | 117 |
| *Culex* (*Melanoconion*) *delpontei* Duret, 1969 | Venezuelan equine encephalitis virus | 118 |
| *Culex* (*Melanoconion*) *pedroi* Sirivanakarn & Belkin, 1980 | Venezuelan equine encephalitis virus | 119 |
| *Haemagogus* (*Conopostegus*) *leucocelaenus* (Dyar & Shannon, 1924) | yellow fever virus | 99,120 |
| *Mansonia* (*Mansonia*) *indubitans* Dyar & Shannon, 1925** | Eastern equine encephalitis virus | 121 |
| *Mansonia* (*Mansonia*) *titillans* (Walker, 1848) | Eastern equine encephalitis virus, West Nile virus | 98,111 |
| *Psorophora* (*Janthinosoma*) *ferox* (von Humboldt, 1819) | Rocio virus, Eastern equine encephalitis virus, West Nile virus, yellow fever virus | 122-124 |
| *Psorophora* (*Janthinosoma*) *lutzii* (Theobald, 1901) | Ilhéus vírus | 125 |
| *Trichoprosopon* (*Trichoprosopon*) *pallidiventer* (Lutz, 1905)*** | Anhembi vírus | 126 |

* Species that have been targeted by mosquito control programs in urban areas

** Species not found naturally infected but whose vector competence has been proven in laboratory studies.

*** Species found naturally infected with arboviruses or wild plasmodia that do not affect humans.

**Table S5. Approximate geographical distances (in km) between the nine parks in the city of São Paulo, Brazil.**

|  | Chico Mendes | Piqueri | Anhanguera | Burle Marx | Santo Dias | Shangrilá | Alfredo Volpi | Previdência |
| --- | --- | --- | --- | --- | --- | --- | --- | --- |
| Ibirapuera | 24.9 | 10.5 | 20.1 | 6.7 | 13.5 | 18.3 | 3.6 | 6.4 |
| Chico Mendes |  | 14.8 | 35.7 | 32.6 | 38.9 | 36.8 | 29 | 31.4 |
| Piqueri |  |  | 22.5 | 18.4 | 24.8 | 27 | 14.2 | 16.4 |
| Anhanguera |  |  |  | 22.8 | 25.5 | 38.1 | 18.7 | 17.1 |
| Burle Marx |  |  |  |  | 5.7 | 15.1 | 4.9 | 5.3 |
| Santo Dias |  |  |  |  |  | 15.1 | 10.6 | 10.1 |
| Shangrilá |  |  |  |  |  |  | 19.2 | 20.6 |
| Alfredo Volpi |  |  |  |  |  |  |  | 2.1 |

**Table S6. Number of mosquitoes by species/trap collected in nine urban parks in the city of São Paulo, Brazil, from March 2011 to February 2012 (Alfredo Volpi Park, Anhanguera Park, Chico Mendes Park, Ibirapuera Park, Santo Dias Park and Shangrilá Park) and August 2012 to July 2013 (Burle Marx Park, Piqueri Park and Previdência Park).**

| **Species** | **Adult traps** | | | | **Larval traps** | | **Total** |
| --- | --- | --- | --- | --- | --- | --- | --- |
|  | Aspirator | CDC (tree canopy) | CDC (ground level) | Shannon | Larval dipper | Suction samplers |  |
| *Aedeomyia squamipennis* |  | 2 |  |  |  |  | 2 |
| *Aedes crinifer* | 2 |  | 1 | 12 |  |  | 15 |
| *Aedes fluviatilis* | 370 | 68 | 278 | 3583 | 32 | 11 | 4342 |
| *Aedes scapularis* | 702 | 195 | 344 | 2964 | 32 |  | 4237 |
| *Aedes serratus* |  |  | 1 | 4 |  |  | 5 |
| *Aedes terrens* | 1 |  |  |  |  |  | 1 |
| *Aedes aegypti* | 34 | 2 | 3 | 2 | 71 | 455 | 563 |
| *Aedes albopictus* | 248 | 4 | 28 | 32 | 486 | 5193 | 5991 |
| *Anopheles fluminensis* | 1 |  | 2 |  | 11 |  | 14 |
| *Anopheles intermedius* |  |  |  |  | 3 |  | 3 |
| *Anopheles evansae* | 2 | 1 |  | 9 | 22 |  | 34 |
| *Anopheles strodei* | 7 | 1 | 1 | 51 | 63 |  | 123 |
| *Anopheles albitarsis* |  |  |  | 1 |  |  | 1 |
| *Coquillettidia chrysonotum/albifera* |  |  |  | 2 |  |  | 2 |
| *Coquillettidia venezuelensis* |  |  | 1 | 1 |  |  | 2 |
| *Coquillettidia juxtamansonia* |  |  |  | 1 |  |  | 1 |
| *Culex acharistus* | 1 |  |  |  |  |  | 1 |
| *Culex bidens* | 193 |  |  | 2 |  |  | 195 |
| *Culex brami* | 1 |  |  |  | 12 |  | 13 |
| *Culex chidesteri* | 397 | 401 | 239 | 40 | 43 | 6 | 1126 |
| *Culex coronator* | 4 |  |  |  |  |  | 4 |
| *Culex declarator* | 1168 | 5 | 5 | 12 | 3 | 31 | 1224 |
| *Culex dolosus* | 2 |  | 1 | 2 | 141 | 15 | 161 |
| *Culex dolosus/eduardoi* | 51 | 2 | 10 | 4 |  |  | 67 |
| *Culex eduardoi* | 3 |  |  |  | 124 | 35 | 162 |
| *Culex gr. Coronator* | 1 | 5 | 1 | 10 | 122 |  | 139 |
| *Culex lygrus* | 62 |  | 1 | 6 |  |  | 69 |
| *Culex nigripalpus* | 1394 | 2000 | 378 | 2377 | 34 |  | 6183 |
| *Culex quinquefasciatus* | 906 | 94 | 91 | 62 | 1799 | 2116 | 5068 |
| *Culex saltanensis* | 19 |  |  |  |  |  | 19 |
| *Culex spp.* | 1377 | 2781 | 1172 | 562 |  |  | 5892 |
| *Culex usquatus* | 1 |  |  |  |  |  | 1 |
| *Culex aliciae* | 1 |  |  |  |  |  | 1 |
| *Culex aureonotatus* | 13 | 9 | 13 | 23 | 1 |  | 59 |
| *Culex bastagarius* | 1 |  |  |  |  |  | 1 |
| *Culex delpontei* | 2 | 2 |  |  |  |  | 4 |
| *Culex intrincatus* | 1 |  |  |  | 31 |  | 32 |
| *Culex cf. maxinocca* | 3 |  |  |  | 23 |  | 26 |
| *Culex pedroi* |  |  |  |  | 2 |  | 2 |
| *Culex ribeirensis* | 1 |  |  | 17 |  |  | 18 |
| *Culex sec. Melanoconion* | 4 | 14 | 5 | 7 | 46 |  | 76 |
| *Culex serratimarge* | 4 |  |  |  | 3 |  | 7 |
| *Culex spp.* |  | 11 |  |  | 4 |  | 15 |
| *Culex vaxus* | 1 | 5 | 45 | 10 | 17 |  | 78 |
| *Culex* gr. Imitator | 9 |  | 1 | 1 |  | 2 | 13 |
| *Culex* gr. Pleuristriatus |  |  |  |  |  | 1 | 1 |
| *Culex imitator imitator* |  |  |  |  | 3 | 444 | 447 |
| *Culex corniger* | 2 | 1 |  |  |  |  | 3 |
| *Haemagogus leucocelaenus* |  |  |  |  |  | 5 | 5 |
| *Limatus durhami* | 15 | 2 | 18 | 4 | 106 | 736 | 881 |
| *Mansonia humeralis* | 1 |  |  |  |  |  | 1 |
| *Mansonia indubitans* | 3 |  |  |  |  |  | 3 |
| *Mansonia titillans* | 2 | 4 | 10 | 57 |  |  | 73 |
| *Mansonia wilsoni* | 15 | 1 | 5 | 12 |  |  | 33 |
| *Psorophora ferox* | 13 |  | 9 | 31 |  |  | 53 |
| *Psorophora lutzii* |  |  |  |  |  | 3 | 3 |
| *Sabethes melanonymphe* |  |  |  | 1 |  |  | 1 |
| *Trichoprosopon pallidiventer/castroi/simile* |  |  |  |  |  | 1 | 1 |
| *Trichoprosopon pallidiventer* |  |  |  |  |  | 8 | 8 |
| *Trichoprosopon* spp. |  | 1 |  | 1 |  |  | 2 |
| *Toxorhynchites* spp. | 1 |  | 1 | 1 | 15 | 108 | 126 |
| *Uranotaenia calosomata* | 1 |  |  |  |  |  | 1 |
| *Uranotaenia lowii* | 4 |  | 2 |  |  |  | 6 |
| *Uranotaenia nataliae* |  |  |  |  | 1 | 1 | 2 |
| *Uranotaenia pulcherrima* |  |  |  |  | 5 |  | 5 |
| *Wyeomyia lutzi* |  |  |  |  |  | 7 | 7 |
| *Wyeomyia oblita* |  |  |  |  |  | 110 | 110 |
| *Wyeomyia davisi* | 1 |  |  |  |  | 72 | 73 |
| *Wyeomyia* cf. *galvaoi* | 3 |  |  |  |  | 53 | 56 |
| *Wyeomyia* sp. | 1 |  |  |  |  |  | 1 |
| *Wyeomyia confusa* | 1 | 3 | 4 | 1 |  |  | 9 |
| *Wyeomyia airosai/ howardi/luteoventralis* | 4 |  |  | 1 |  |  | 5 |
| *Wyeomyia serratoria* |  |  |  |  |  | 50 | 50 |
| *Wyeomyia* spp. | 2 |  |  | 1 |  | 11 | 14 |
| **Total no. of specimens** | 7056 | 5614 | 2670 | 9907 | 3255 | 9474 | 37972 |
| **No. of observed species/taxa** | 51 | 25 | 29 | 37 | 29 | 24 | 73 |


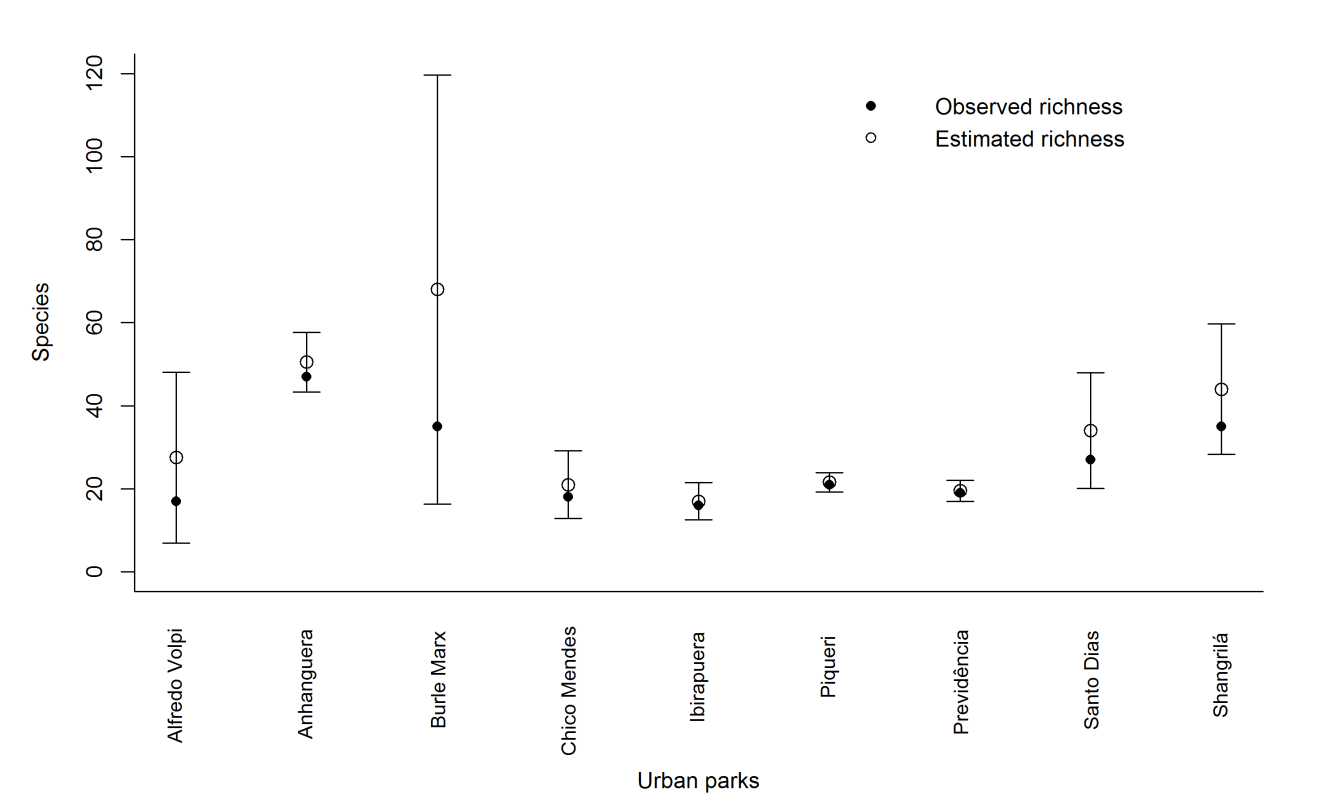


**Fig S1.** Estimated (Chao 1, 95% CI) and observed mosquito richness for the nine parks in the city of São Paulo, Brazil.


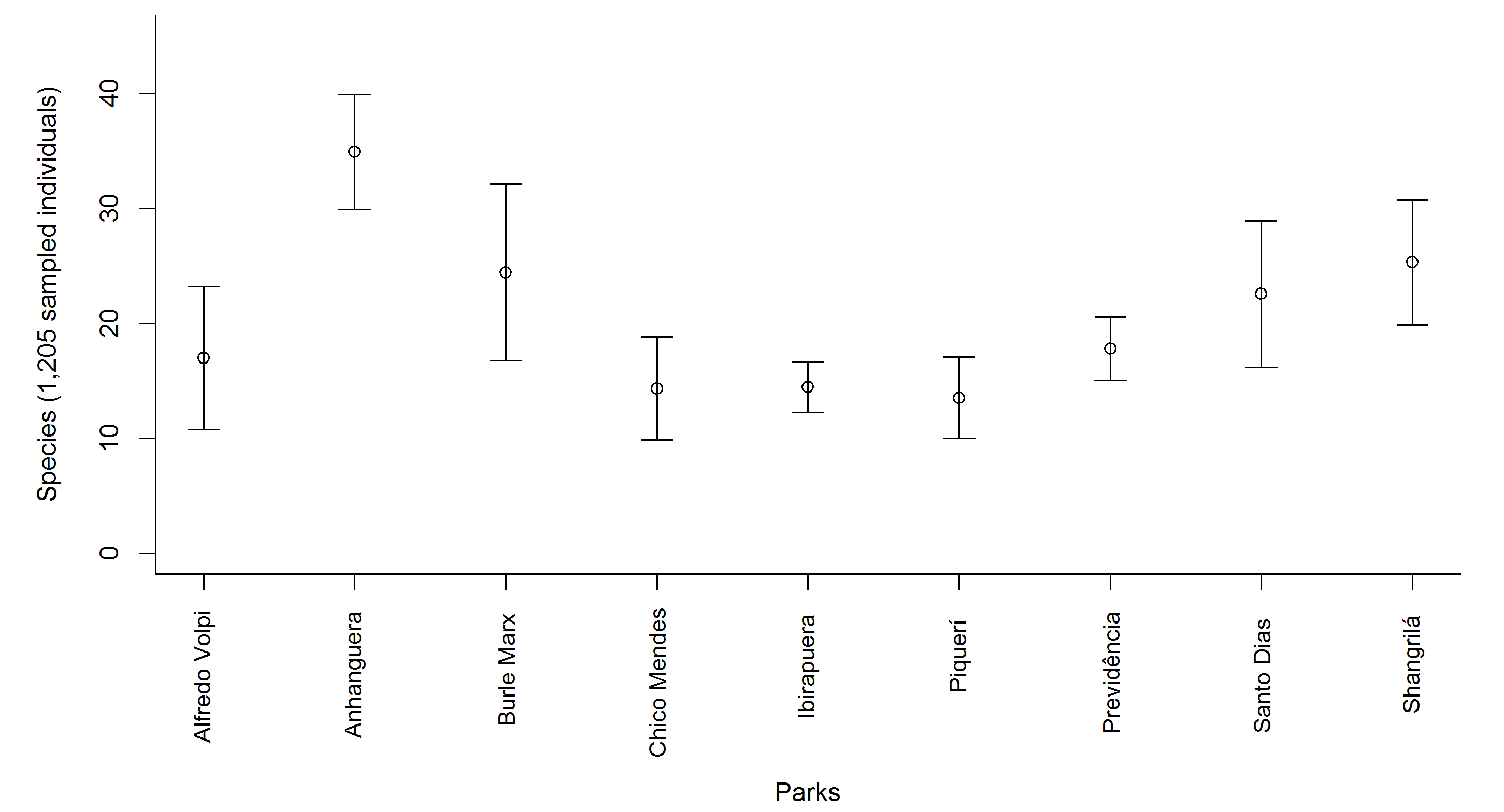


**Fig S2.** Comparative mosquito richness based on 1,000 random resamplings of 1,205 individuals in each urban park. The number 1,205 refers to the total number of mosquitoes collected in Alfredo Volpi Park, which had the lowest abundance of the parks studied. The error bars represent the 95% confidence interval.


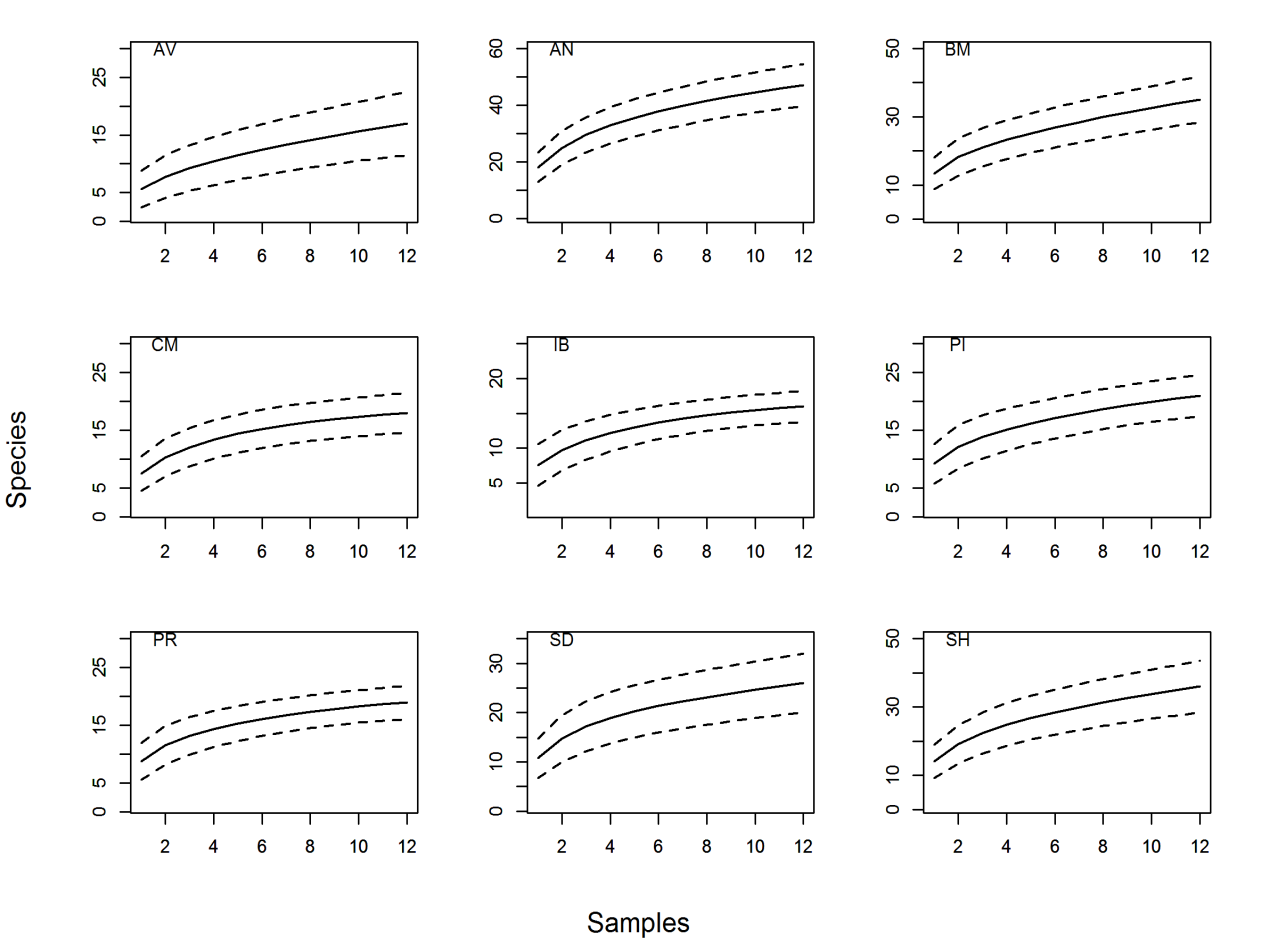


**Fig S3.** Species accumulation curves (1,000 randomizations without replacement and 95% CI) for the nine parks in the city of São Paulo, Brazil, from which specimens were collected. AV - Alfredo Volpi, AN – Anhanguera, BM - Burle Marx, CM – Chico Mendes, IB – Ibirapuera, PI – Piqueri, PR – Previdência, SD – Santo Dias, SH – Shangrilá.


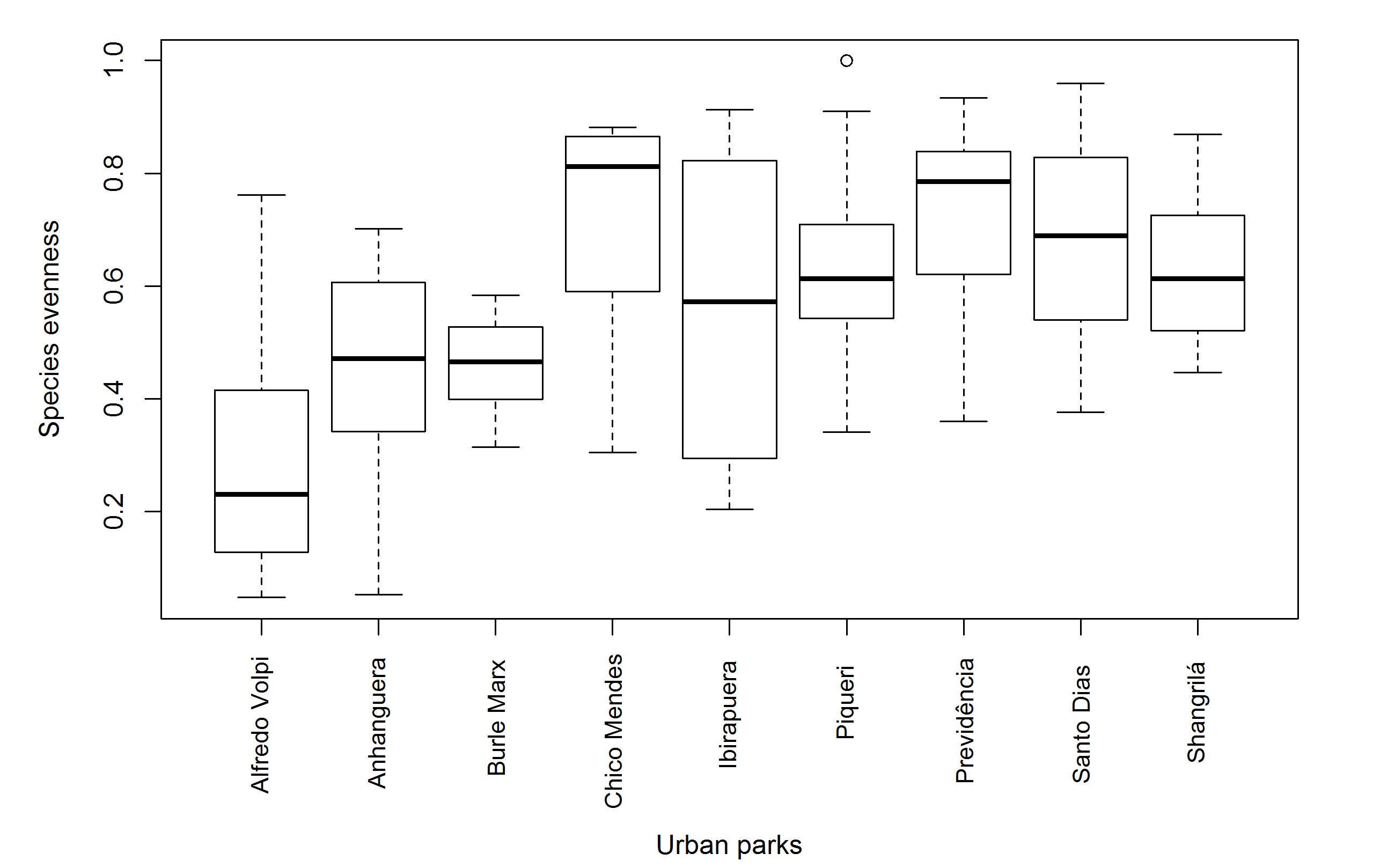


**Fig S4.** Boxplot showing monthly variations in the evenness of mosquito assemblages from nine urban parks in the city of São Paulo during the study period. Pielou's evenness index (J) was used and only adult forms were considered.

**Additional References**

91. Eastwood, G. *et al.* Enzootic arbovirus surveillance in forest habitat and phylogenetic characterization of novel isolates of Gamboa virus in Panama. *Am. J. Trop. Med. Hyg.* **94,** 786–793 (2016).

92. Davis, N. C. & Shannon, R. C. Studies on yellow fever in South America. Attempts to transmit the virus with certain Aedine and Sabethine mosquitoes and with Triatomas (Hemiptera). *Am. J. Trop. Med. Hyg.* **11,** 21–29 (1931).

93. de Camargo, M. V., Cônsoli, R. A., Williams, P. & Krettli, A. U. Factors influencing the development of *Plasmodium gallinaceum* in *Aedes fluviatilis*. *Memorias do Instituto Oswaldo Cruz* **78,** 83–94 (1983).

94. Silva, J.B.L. *et al*. Wolbachia and dengue virus infection in the mosquito *Aedes fluviatilis* (Diptera: Culicidae). *PLoS One* ***12,*** (2017).

95. Spence, L., Anderson, C. R. & Downs, W. G. Melao virus, a new agent isolated from Trinidadian mosquitoes. *Am J Trop Med Hyg.* **11,** 687–690 (1962).

96. Mitchell, C. J., Forattini, O. P. & Miller, B. R. Vector competence experiments with Rocio virus and three mosquito species from the epidemic zone in Brazil. *Rev. Saude Publica* **20,** 171–177 (1986).

97. Mitchell, C. J. & Forattini, O. P. Experimental Transmission of Rocio Encephalitis Virus by *Aedes Scapularis* (Diptera: Culicidae) from the Epidemic Zone in Brazil. *J. Med. Entomol.* **21,** 34–37 (1984).

98. Walter Reed Biosystematics Unit. Southcom medically important arthropods. *Walter Reed Army Institute of Research* (2016). Available at: http://www.wrbu.org/aors/southcom.html.

99. Cardoso, J. da C. *et al.* Yellow fever virus in *Haemagogus leucocelaenus* and *Aedes serratus* mosquitoes, Southern Brazil, 2008. *Emerg. Infect. Dis.* **16,** 1918–1924 (2010).

100. Chouin-Carneiro, T. *et al.* Differential Susceptibilities of *Aedes aegypti* and *Aedes albopictus* from the Americas to Zika Virus. *PLoS Negl. Trop. Dis.* **10,** (2016).

101. Vega-Rua, a., Zouache, K., Girod, R., Failloux, a.-B. & Lourenco-de-Oliveira, R. High Level of Vector Competence of *Aedes aegypti* and *Aedes albopictus* from Ten American Countries as a Crucial Factor in the Spread of Chikungunya Virus. *J. Virol.* **88,** 6294–6306 (2014).

102. Gerhardt, R. R. *et al.* First isolation of La Crosse virus from naturally infected *Aedes albopictus*. *Emerg. Infect. Dis.* **7,** 807–811 (2001).

103. Holick, J., Kyle, A., Ferraro, W., Delaney, R. R. & Iwaseczko, M. Discovery of *Aedes albopictus* infected with west nile virus in southeastern Pennsylvania. *J. Am. Mosq. Control Assoc.* **18,** 131 (2002).

104. Neves, A. *et al.* Malaria outside the Amazon region: Natural *Plasmodium* infection in anophelines collected near an indigenous village in the Vale do Rio Branco, Itanhaém, SP, Brazil. *Acta Trop.* **125,** 102–106 (2013).

105. De Oliveira-Ferreira, J., Lourenco-de-Oliveira, R., Teva, A., Deane, L. M. & Daniel-Ribeiro, C. T. Natural malaria infections in anophelines in Rondonia state, Brazilian Amazon. *Am. J. Trop. Med. Hyg.* **43,** 6–10 (1990).

106. Duarte, A. M. R. C. *et al.* Natural infection in anopheline species and its implications for autochthonous malaria in the Atlantic Forest in Brazil. *Parasit. Vectors* **6,** 58 (2013).

107. Velasquez, G. Bionomics, Ecology and Medical Importance of *Coquillettidea* (*Rhynchotaenia*) *venezuelensis* Theobald, 1912 (Diptera: Culicidae). *Saber, Univ. Oriente, Venez.* **26,** 105–113 (2014).

108. Pauvolid-Corrêa, A. *et al.* Nhumirim virus, a novel flavivirus isolated from mosquitoes from the Pantanal, Brazil. *Arch. Virol.* **160,** 21–27 (2015).

109. Vasconcelos, P. F. d. C. *et al.* Epidemiologia das encefalites por arbovírus na Amazônia Brasileira. *Revista do Instituto de Medicina Tropical de Sao Paulo* **33,** 465–476 (1991).

110. Alto, B. W., Connelly, C. R., O’Meara, G. F., Hickman, D. & Karr, N. Reproductive Biology and Susceptibility of Florida *Culex coronator* to Infection with West Nile Virus. *Vector Borne Zoonotic Dis.* **14,** 606–14 (2014).

111. Centers for Disease Control and Prevention*.* Mosquito species in which West Nile virus has been detected, United States, 1999-2012. *CDC* **7,** 722–725 (2012).

112. Monath TP, Cropp CB, Bowen GS, Kemp GE, Mitchell CJ, G. J. Variation in Virulence for Mice and Origin Monkeys Virus Among Strains St . Louis Encephalitis of Different Origin. *Am. J. Trop. Med. Hyg.* **29,** 948–962 (1980).

113. Turell, M. J. *et al.* An Update on the Potential of North American Mosquitoes (Diptera: Culicidae) to Transmit West Nile Virus. *J. Med. Entomol.* **42,** 57–62 (2005).

114. Ezenwa, V. O., Godsey, M. S., King, R. J. & Guptill, S. C. Avian diversity and West Nile virus: testing associations between biodiversity and infectious disease risk. *Proc. R. Soc. B Biol. Sci.* **273,** 109–117 (2006).

115. Dhanda, V. *et al.* Japanese encephalitis virus infection in mosquitoes reared from field-collected immatures and in wild-caught males. *Am. J. Trop. Med. Hyg.* **41,** 732–736 (1989).

116. Da Silva Heinen, L. B. *et al.* Saint Louis encephalitis virus in Mato Grosso, Central-Western Brazil. *Rev. Inst. Med. Trop. Sao Paulo* **57,** 215–220 (2015).

117. Lourenço-de-Oliveira, R. & de Castro, F. A. *Culex saltanensis* Dyar, 1928--natural vector of *Plasmodium juxtanucleare* in Rio de Janeiro, Brazil. *Mem. Inst. Oswaldo Cruz* **86,** 87–94 (1991).

118. Mitchell, C. J. *et al.* Arbovirus investigations in Argentina, 1977-1980. II. Arthropod collections and virus isolations from Argentine mosquitoes. *Am. J. Trop. Med. Hyg.* **34,** 945–955 (1985).

119. Vasconcelos, P. F. *et al.* Isolations of yellow fever virus from *Haemagogus leucocelaenus* in Rio Grande do Sul State, Brazil. *Trans. R. Soc. Trop. Med. Hyg.* **97,** 60–62 (2003).

120. Ferro, C. *et al*. Natural enzootic vectors of Venezuelan equine encephalitis virus, Magdalena Valley, Colombia. *Emerg. Infect. Dis.* **9**, 49-54 (2003)

121. Turell, M. J. *et al.* Vector competence of Peruvian mosquitoes (Diptera: Culicidae) for epizootic and enzootic strains of Venezuelan equine encephalomyelitis virus. *J. Med. Entomol.* **37,** 835–9 (2000).

122. de Souza Lopes, O., de Abreu Sacchetta, L., Francy, D. B., Jakob, W. L. & Calisher, C. H. Emergence of a new arbovirus disease in Brazil. III. Isolation of Rocio virus from *Psorophora Ferox* (Humboldt, 1819). *Am. J. Epidemiol.* **113,** 122–5 (1981).

123. Kulasekera, V. L. *et al.* West Nile virus infection in mosquitoes, birds, horses, and humans, Staten Island, New York, 2000. *Emerg. Infect. Dis.* **7,** 722–725 (2001).

124. Moreno, E. S. *et al.* Reemergence of yellow fever: detection of transmission in the State of Sao Paulo, Brazil, 2008. *Rev. Soc. Bras. Med. Trop.* **44,** 290–296 (2011).

125. De Rodaniche, E. & Galindo, P. Ecological observations on Ilhéus virus in the vicinity of Almirante, Republic of Panama. *Am. J. Trop. Med. Hyg.* **12,** 924–928 (1963).

126. De Souza Lopes, O., De Abreu Sacchetta, L., Fonseca, I. E. M. & Lacerda, J. P. G. Bertioga (Guama group) and Anhembi (Bunyamwera group), two new arboviruses isolated in São Paulo, Brazil. *Am. J. Trop. Med. Hyg.* **24,** 131–134 (1975).
